# Complete genome sequence of *Pseudomonas aeruginosa* Y010, a taro rhizosphere strain producing potent antimicrobial agents

**DOI:** 10.1128/mra.00668-24

**Published:** 2024-08-20

**Authors:** Yongqi Wen, Huiluo Cao, Zhifeng Mo, Hanshu Fang, Zeling Xu

**Affiliations:** 1 Guangdong Province Key Laboratory of Microbial Signals and Disease Control, Integrative Microbiology Research Centre, South China Agricultural University, Guangzhou, China; 2 State Key Laboratory of Green Pesticide, College of Plant Protection, South China Agricultural University, Guangzhou, China; 3 Department of Microbiology, Li Ka Shing Faculty of Medicine, The University of Hong Kong, Hong Kong SAR, China; The University of Arizona, Tucson, Arizona, USA

**Keywords:** *P. aeruginosa*, *Dickeya*, biocontrol, antimicrobial agents

## Abstract

*Pseudomonas aeruginosa* Y010, isolated from the taro rhizosphere, exhibits great antagonistic abilities against *Dickeya* strains that cause soft-rot and blackleg diseases of plants by producing potent antimicrobial agents. The complete genome of Y010 was sequenced and annotated, which is 6,415,628 bp in length with 66.39% GC content.

## ANNOUNCEMENT

**Fig 1:**
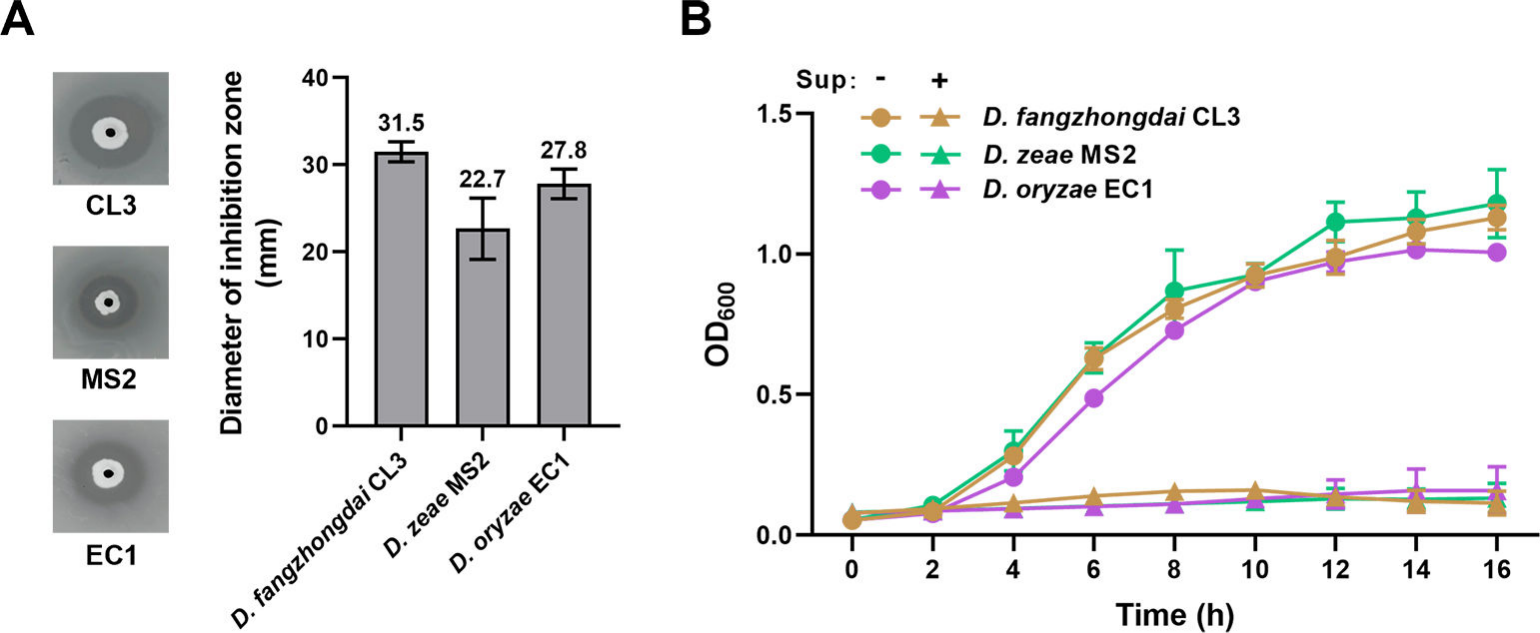
(**A**) Antagonistic activities of *P. aeruginosa* Y010 against *Dickeya* pathogens (*D. fangzhongdai* CL3, *D. zeae* MS2, and *D. oryzae* EC1) determined using spot-on-lawn assay. Diameters of inhibition zones as shown in the left panel were measured and are presented in the right panel. (**B**) Growth of the *Dickeya* pathogens in the presence or absence of the Y010 culture supernatant (Sup).

The *Dickeya* genus is one of the most destructive phytopathogens (1). To screen biocontrol bacterial strains against plant diseases caused by *Dickeya* strains, we collected soil samples at the taro rhizosphere (23°09'38.6"N and 113°21'31.9"E) in Guangzhou, Guangdong, People’s Republic of China. Ten grams of the soil sample was added in 90 mL of sterilized Milli-Q water and shaken for 30 minutes. Next, 100 µL of the suspension was serially diluted and spread on lysogeny broth (LB) agar plates. Bacterial strains with single colonies were obtained after the plates were incubated at 28°C for 48 hours. The antagonistic ability of isolated strains against *Dickeya* strains was determined using the spot-on-lawn assay (2). Y010 was obtained owing to its great antagonistic effect and its production of potent antimicrobial agents against various *Dickeya* strains, including *D. fangzhongdai* CL3, *D. zeae* MS2, and *D. oryzae* EC1 (Fig. 1). According to the comparison of the 16S rRNA sequences, the strain Y010 showed a 100% similarity to members within the species *Pseudomonas aeruginosa*.

A single colony of *P. aeruginosa* Y010 was inoculated in 10 mL of the LB broth at 28°C with 220 rpm agitation for 20 hours. Genomic DNA was extracted from the bacterial culture using the EasyPure Genomic DNA Kit (TransGen, China) to prepare both short-read and long-read libraries. A short-read library was prepared with the Nextera XT DNA Library Preparation Kit (Illumina, USA) and sequenced using an Illumina NovaSeq 6000 platform with 150-bp paired-end reads (Novogene, China), which contained 7,519,498 pairs of reads with a total length of 2.26 gigabase (Gb). A long-read library was prepared with Ligation Sequencing Kit V14 (SQK-LSK114) without size selection and shearing and sequenced using a flow cell (R10.4.1) and Nanopore MinION sequencer Mk1B (Oxford Nanopore Technologies, UK), which generated 56,049 reads with a total length of 676.39 million bp (Mb) (*N_50_*, 18,737 bp and mean length, 12,067.8 bp) using Guppy v4.2.2 for base-calling. Quality-based filtering and adapter trimming were performed using Trimmomatic v0.39 (3) and NanoFilt v2.8.0 (4), respectively. The genome was hybrid-assembled using Unicycler v0.50 (5). The quality of the assembled genome was evaluated using QUAST v5.0.2 (6). Circulator v1.5.5 was used to circularize and rotate the genome with the *dnaA* gene of *P. aeruginosa* PAO1 (GenBank: AE004091) as the reference (7). The NCBI Prokaryotic Genome Annotation Pipeline (Version 6.7 March 2024) was used for gene annotation. rRNA, non-coding RNAs, and tRNAs were annotated by Rfam (v.14.4) (8), Infernal (v.1.1.5) (9), and tRNAscan-SE (v2.0.12), respectively (10). Default parameters were used for all the abovementioned software.

Collectively, the assembly yielded a single genome. The circular genome size of Y010 is 6,415,628 bp in length with 66.39% GC content and 367 x average coverage depth. A total of 5,885 genes were predicted in the Y010 genome, including 5,775 coding gene sequences (CDS), 31 pseudogenes, 12 rRNAs, 63 tRNAs, and four ncRNAs (Table 1). Two prophages were identified using PHASTEST (11), and three CRISPR arrays were revealed using CRISPRCasFinder (12). Based on PubMLST (13), multiple locus sequence type (MLST) of Y010 was assigned to MLST633.

**TABLE 1:** Genomic information for *P. aeruginosa* Y010

Bacterial strain	*P. aeruginosa* Y010
Total length (bp)	6,415,628
% GC content	66.39
Genes	5,885
Proteins	5,775
rRNA	12
tRNA	63
ncRNA	4
Pseudogenes	31
CRISPR arrays	3
Prophages	2

## Data Availability

The genomic data of *P. aeruginosa* Y010 were deposited in the NCBI under the BioProject accession number PRJNA1116115, the BioSample accession number SAMN41523228, and the GenBank accession number CP158050. Sequence Read Archive (SRA) accession numbers for Illumina and Nanopore reads are SRX24924469 and SRX24924471, respectively.
